# Associations between overweight and mental health problems among adolescents, and the mediating role of victimization

**DOI:** 10.1186/s12889-019-6832-z

**Published:** 2019-05-21

**Authors:** Cornelia Leontine van Vuuren, Gusta G. Wachter, René Veenstra, Judith J. M. Rijnhart, Marcel F. van der Wal, Mai J. M. Chinapaw, Vincent Busch

**Affiliations:** 1 Department of Epidemiology, Health Promotion and Healthcare Innovation, Public Health Service (GGD) Amsterdam, Nieuwe Achtergracht 100, 1018 WT Amsterdam, the Netherlands; 2 Department of Sociology, Faculty of Behavioral and Social Sciences, University of Groningen, Grote Kruisstraat 2/1, 9712 TS Groningen, the Netherlands; 3 Department of Epidemiology and Biostatistics, Amsterdam Public Health Research Institute, Amsterdam UMC, location VU University Medical Center, P.O. BOX 7057, 1007 MB Amsterdam, the Netherlands; 4 Department of Public and Occupational Health, Amsterdam Public Health Research Institute, Amsterdam UMC, Vrije Universiteit Amsterdam, P.O. BOX 7057, 1007 MB, Amsterdam, the Netherlands

**Keywords:** Overweight, Obesity, Mental health problems, Bullying victimization, Youth

## Abstract

**Background:**

Evidence has not been conclusive on whether adolescent overweight is associated with mental health, possibly caused by indirect, yet untested associations. Therefore, the purpose of this study was to examine the association between overweight or obesity and mental health problems among adolescents, and to determine whether victimization plays a mediating role in these associations.

**Methods:**

Self-reported data on mental health and victimization and objectively measured Body Mass Index data were used, using three cohorts (2010–2011 until 2012–2013) and an interval between the measurement waves of two years later. We performed a multi-level mediation analysis with a two-level structure to incorporate the clustering of the measurements within individuals. The study population consisted of 13,740 secondary school students, 13–14 years old at the first measurement moment, in Amsterdam, the Netherlands.

**Results:**

Compared to their normal-weight peers, adolescents with overweight or obesity reported psychosocial problems and suicidal thoughts more often. Victimization was a significant mediator in the relationship between having overweight, and psychosocial problems (indirect effect OR: 2.3; 95% CI 1.5, 3.7 and direct effect OR: 1.4; 95% CI 1.2, 1.7) or suicidal thoughts (indirect effect OR: 2.1; 95% CI 1.4, 3.2 and direct effect OR: 1.3; 95% CI 1.1, 1.5). The associations between obesity, and psychosocial problems (indirect OR: 6.2; 95% CI 2.8, 14.7 and direct effect OR: 1.4; 95% CI 1.0, 2.0), or suicidal thoughts (indirect OR: 4.5; 95% CI 2.3, 9.1 and direct effect OR: 1.5; 95% CI 1.1, 2.0) were even stronger.

**Conclusions:**

Overweight and obesity were significantly associated with mental health problems in adolescents, and victimization played a mediating role in this association. Victimization and mental health should be integrated into prevention programs that address healthy weight development. Moreover, overweight should be given more attention in programs to prevent victimization and promote adolescent mental health.

**Electronic supplementary material:**

The online version of this article (10.1186/s12889-019-6832-z) contains supplementary material, which is available to authorized users.

## Background

Adolescent overweight is a widespread phenomenon [1, 2]. In 2016, globally around 17% of teenagers had overweight, including obesity [3]. In the Netherlands, around 15% of the 14-and 16-years-olds have overweight [4] and in Amsterdam the prevalence is around 20% [5–7]. Adolescent overweight, besides affecting physical health, may negatively impact mental health. However, evidence has not been conclusive on whether adolescent overweight is associated with psychosocial problems and suicidal thoughts. Several studies have found an association with mental health problems, such as depression, suicide ideation and attempts, anxiety, behavioral problems, low self-esteem and poor self-image [8–14], whereas other studies found no evidence of such associations [15–18].

These inconsistent findings might be caused by indirect associations between both overweight and obesity and mental health problems that have not been tested [19]. A possible mediator is victimization [20–22]. Being overweight is not in line with the prevailing Western cultural norms and ideals about physical appearance [23]. Adolescents with overweight stand out negatively and often develop a poor self-image, which increases their risk of being bullied by peers [24–32]. Although some studies mentioned the possibility of victimization as a mediator between body weight and mental health (i.e. depression, anxiety, emotional problems, self-esteem, body dissatisfaction and eating disorders) [14, 33, 34], only a few have conducted mediation analyses [35–37]. However, these studies used small samples, cross-sectional designs or young age groups.

This knowledge gap needs to be addressed to acquire the information needed to design effective interventions promoting healthy mental health development in adolescence. We therefore examined the association of overweight (including obesity) and obesity, with psychosocial problems and suicidal thoughts, including an analysis of the potential mediating role of being a victim of bullying. We used a combination of cross-sectional and repeated measurements (two waves) for a subset of respondents from a large, population-representative sample of adolescents in Amsterdam, the Netherlands.

## Methods

### Sample and materials

All secondary schools in Amsterdam require routine health assessments of their students by the Amsterdam Public Health Service (GGD). This assessment includes a clinical interview and completion of a self-reported electronic health questionnaire, before the interview. The data in this study were obtained from this questionnaire and from the Digital Child Health Care Registry (DCHCR), which administers the records of these physical examinations. Both data sources are part of the Youth Health Monitor (YHM) of the GGD and are described in more detail below. We combined three cohorts from the YHM. The first data wave was collected in school years 2010–2011 (cohort 1), 2011–2012 (cohort 2), and 2012–2013 (cohort 3). At that time the students were in the eighth grade, the second year of Dutch secondary education when students are on average 14 years old. Two years later, in the tenth grade, the second wave was collected for each cohort.

The health questionnaire contained questions about sociodemographic characteristics, lifestyles and physical and mental health problems. These questionnaires were completed during school hours in an exam room under supervision of a teacher and a school nurse of the GGD. To avoid socially desirable answers, the school nurse explained to the students that their answers were confidential and were known only to the school nurse or possibly a physician. Before data collection, information letters were sent to parents and students. A passive informed consent procedure was used, so students and their parents could decide not to complete the questionnaire. The response rate on the health questionnaire was around 90% annually. The most common reason for non-response was absence of the student on the day the questionnaire was given. Based on Little’s MCAR Test in SPSS, we determined that missing data were missing completely at random (Chi-Square = .95, DF = 2, *p* = .62).

In addition, we used objectively assessed body height and weight, and sociodemographic variables from the DCHCR. The DCHCR is a standard government data registry system that includes information on ≥95% of Amsterdam’s youth. At their child’s first routine health assessment, parents are asked for their permission to use data from the DCHCR for research purposes by the GGD.

**Fig. 1:**
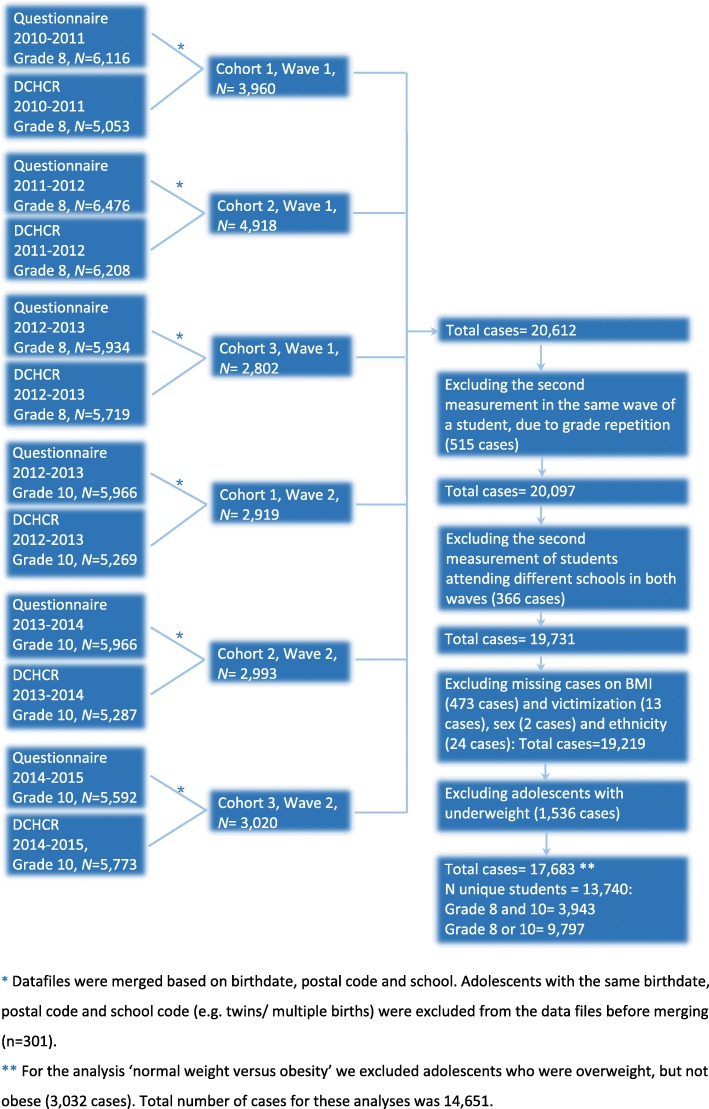
Procedure for merging data from the electronic questionnaire and the Digital Child Health Care Registry between school years 2010–2011 and 2014–2015

We aggregated and anonymised the data from the questionnaires and the DCHCR. To answer our research questions, data from the questionnaires and DCHCR were merged (Fig. 1).

### Measures

#### Mental health problems

Two aspects of mental health were assessed in our study: psychosocial problems and suicidal thoughts.

Psychosocial problems were assessed by the Strengths and Difficulties Questionnaire (SDQ), which is a 25-item, worldwide used screening questionnaire that asks students to report on their behaviors and emotions in the past six months. The items are distributed across five scales of five items each: emotional symptoms, conduct problems, hyperactivity/inattention, peer relationship problems and pro-social behavior. Items are scored on a three-point Likert scale (‘not true’, ‘somewhat true’, ‘certainly true’, scored 0–2). A total difficulties score was calculated as the sum of scores of the first four subscales. We concentrate on adolescents with a relatively high (elevated) score as an indicator of serious psychosocial problems which needed further investigation or action. To determine subgroups, the scores were dichotomized (elevated score: total difficulties scale > 15) based on statistical analyses (ROC-analyses with elevated ASEBA scores as the criterion) and clinical practice (minimizing the chance to miss true cases) [38].

Previous studies have shown good validity and reliability of the SDQ total difficulties score self-report version in Dutch adolescents. Due to concerns regarding the reliability of the subscales, it is recommended to use only the total difficulties score as an indicator of psychosocial problems [38–40].

Suicidal thoughts were assessed with the following question: “During the past 12 months*,* have you ever seriously thought about ending your life?”, which is similar to the Youth Risk Behavior Questionnaire Survey from the USA [41]. The response categories were “never”, “rarely”, “sometimes”, “often”, “very often” and, similar to other studies on the topic, dichotomized into “no” (never) or “yes” (other categories)^.^ [41, 42]. Acceptable test-retest reliability of this measure was previously demonstrated (Kappa = .74) [41].

#### Victimization

We measured victimization by asking “How many times have you been bullied in the past three months at school?” By adding a frequency component to this question the repeated nature of the behavior was included. Respondents could choose from five response categories: “never”, “less than twice per month”, “two or three times per month”, “about once a week”, “several times a week”. This operationalization was based on the Olweus Bully Victim Score [43]. We dichotomized this variable into being bullied at least twice a month or not, in line with Solberg and Olweus [43, 44]. Adequate validity and reliability have been demonstrated [44].

#### Weight status

During their routine health assessment, all participants’ body weight and height were measured by a medical assistant and recorded to one decimal place. If participants were weighted with their clothes on, the medical assistant reduced their recorded weight by 0.5 to 1.0 k, depending on the clothes the student was wearing. Both height and weight were measured without shoes. We distinguished three Body Mass Index (BMI) groups: normal weight, overweight, and obesity based on International Obesity Task Force (IOTF) BMI cutoffs for children [45]. In Amsterdam, where the overweight prevalence is around 20%, normal weight is the prevailing norm among adolescents [5–7]. For the analysis we created two dummies for weight status: “overweight including obesity versus normal weight” and “obese versus normal weight”. As used in this paper, ‘overweight’ refers to overweight including obesity. Students with underweight were excluded from the analysis (Fig. 1).

#### Sociodemographics

Sex and ethnicity as registered in the DCHCR were used. In accordance with the definition of Statistics Netherlands, we considered a student to be of non-Dutch ethnic background when at least one parent was foreign-born [46]. We categorized ethnicity into the five largest groups in the Netherlands: Dutch, Surinamese, Turkish, Moroccan and other.

### Statistical analyses

We analyzed the association between overweight and obesity, and adolescents’ mental health, and whether this was mediated by victimization. For some students we had data available for either T0 or T1, and for others we had data available for both T0 and T1. We performed a multi-level mediation analysis with a two-level structure to incorporate the clustering of the measurements within individuals. As our study was observational, there was already a relationship between the variables established that we measured at time point one. Furthermore, our data was collected with two years between the first and second wave. As it was more likely that overweight, victimization and mental health influenced each other in a relatively short period of time [10, 20], the contemporaneous effects were a better representation of the relationships between the variables than the lagged relationships. By analyzing the data with a multilevel model we used all available information while taking into account the correlation among the repeated measures for part of the group. By using two waves of data for some of the pupils we get insight in both the within- and between-person effect, i.e. the effect estimates in our models were based on a combined within- and between-subject interpretation [47, 48]. We checked whether schools should be added as a third level in our analyses, but because the intra-class correlation was very small (.02) this level was disregarded. Class was not added as a cluster level, because students were in different classes in T0 and T1. Moreover, in the Dutch education system, education is given on the basis of subject clusters, with a general component that is the same for all pupils and an elective component. Furthermore, we tested for significant differences in the indirect effects between boys and girls. Sex was not a moderator (*p* < 0.10).

We used a two-step approach to analyze mediation. First, we examined the original total effect of overweight (independent variable) on psychosocial problems and suicidal thoughts (outcome variables). Second, we fitted a model in which overweight was related to victimization (*a* coefficient) and a model in which overweight and victimization were related to psychosocial problems or suicidal thoughts (direct effect and *b* coefficient respectively), adjusted for sex and ethnicity. Mediation was calculated as the product of the *a* and *b* coefficients. Confidence intervals *(CI)* for the indirect effect estimates (a*b) were based on Monte Carlo simulations [49] to account for the skewed distribution of the indirect effect.

Based on previous studies [36, 37] and on a sensitivity analysis, we hypothesized that adolescents with a more norm-deviating physical appearance, referring to adolescents with obesity, would be especially vulnerable to victimization and thereby to mental health problems. To test this hypothesis, we repeated all analyses for adolescents with obesity in comparison to their normal weight peers. All models were fitted using Mplus version 7 [50] using the Full Information Maximum Likelihood for handling missing data.

## Results

### Population characteristics

In total, 13,740 unique students were included in our study. From 3943 students we gathered information in grade 8 and grade 10, and from 9797 students we had one measurement moment from either grade 8 or grade 10 (Fig. 1). At T0 the mean age of the participants was 14 years, 47% were male and 40% of the students were of Dutch ethnic origin. Also at T0, 18% of the students had overweight and 6% obesity, 7% of the students had been bullied at school in the past three months, 11% had psychosocial problems and 17% had suicidal thoughts during the past 12 months. More details on population characteristics are presented in Table 1.

**Table 1:** Characteristics and distribution of study variables of eighth and tenth grade students participating in the Amsterdam Youth Health Monitor between school years 2010–2011 and 2014–2015

	Grade 8	Grade 10	Total
Participants (n)	10,009	7674	17,683
Mean age (years)	14.01	15.96	14.86
Sex (%)			
Boys	47.4	45.6	46.6
Girls	52.6	54.4	53.4
Ethnicity (%)			
Dutch	39.7	40.4	40.0
Surinamese	10.8	10.9	10.8
Turkish	9.0	9.2	9.0
Moroccan	15.8	15.3	15.6
Other	24.7	24.3	24.5
Overweight, excl. Obese (%)	18.0	16.1	17.1
Obese, excl. Overweight (%)	5.5	4.7	5.2
Bullying victimization (%)	7.1	1.9	4.8
Psychosocial problems (%)	10.6	8.2	9.5
Suicidal thoughts (%)	16.5	9.6	13.5

### Psychosocial problems

**Fig. 2:**
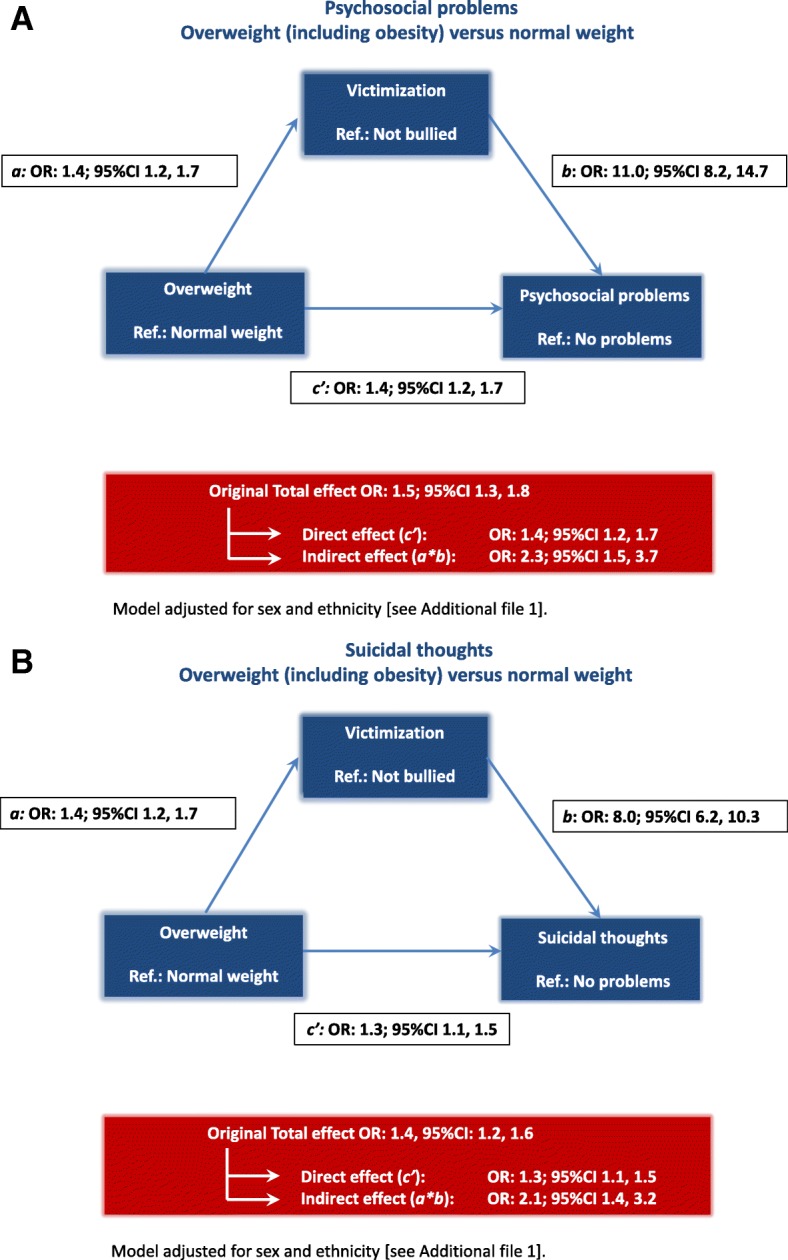
**a** Direct and indirect effects (through victimization) of having overweight on psychosocial problems, adjusted for sex and ethnicity, Amsterdam Youth Health Monitor between school years 2010–2011 and 2014–2015. **b** Direct and indirect effects (through victimization) of having overweight on suicidal thoughts, adjusted for sex and ethnicity, Amsterdam Youth Health Monitor between school years 2010–2011 and 2014–2015

Compared to their normal-weight peers, adolescents with overweight reported psychosocial problems more often (original total effect OR: 1.5; 95% CI 1.3, 1.8) and victimization more often (*a* coefficient OR: 1.4; 95% CI 1.2, 1.7). Adolescents who reported being bullied more often also reported psychosocial problems (*b* coefficient OR: 11.0; 95% CI 8.2, 14.7). Victimization was a significant mediator in the relationship between having overweight and psychosocial problems (indirect OR: 2.3; 95% CI 1.5, 3.7). However, victimization only partly mediated the original association between having overweight and psychosocial problems; for psychosocial problems the direct effect also remained significant (direct effect OR: 1.4; 95% CI 1.2, 1.7) after the mediation effect of victimization was added to the model. This is illustrated in Fig. 2a and Additional file 1.

**Fig. 3:**
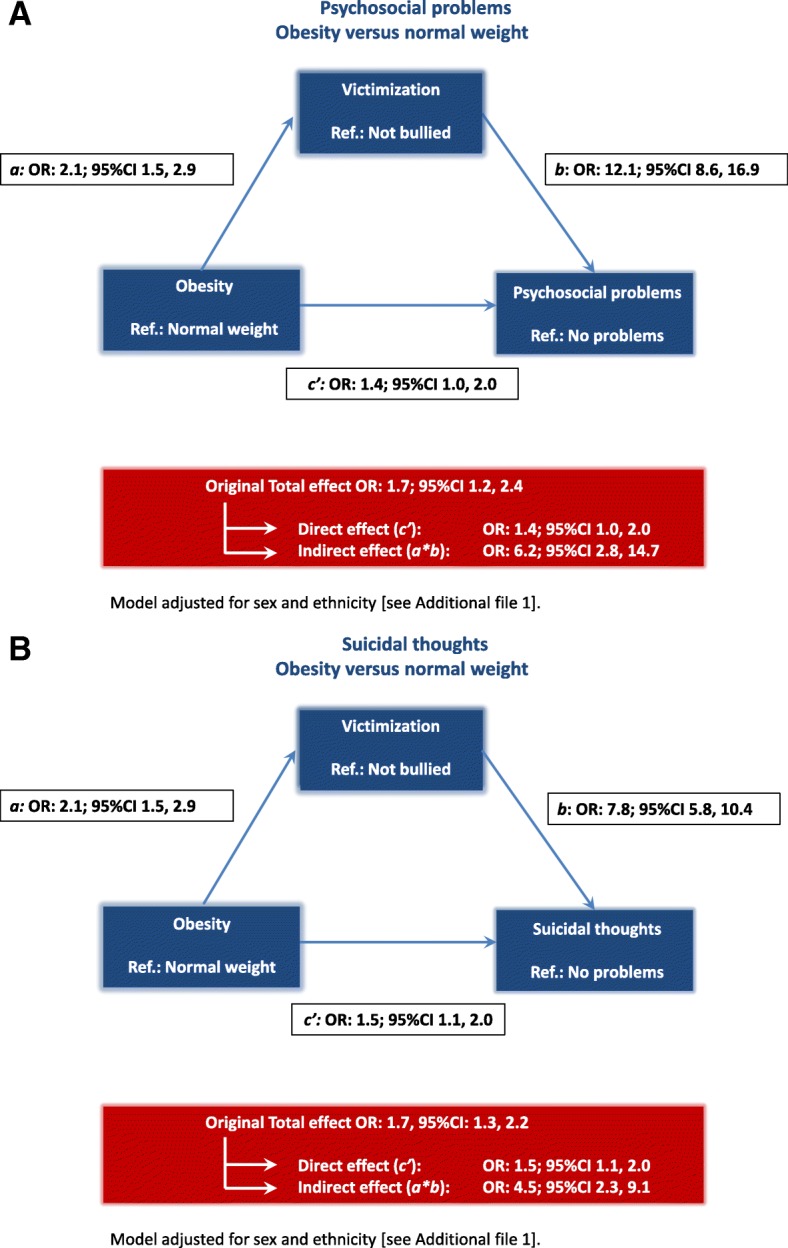
**a** Direct and indirect effects (through victimization) of having obesity on psychosocial problems, adjusted for sex and ethnicity, Amsterdam Youth Health Monitor between school years 2010–2011 and 2014–2015. **b** Direct and indirect effects (through victimization) of having obesity on suicidal thoughts, adjusted for sex and ethnicity, Amsterdam Youth Health Monitor between school years 2010–2011 and 2014–2015

When we compared adolescents with obesity to their normal weight peers, the studied associations were even stronger. Although the direct effect remained comparable (OR: 1.4; 95% CI 1.0, 2.0), the indirect effect was larger (OR: 6.2; 95% CI 2.8, 14.7). This is illustrated in Fig. 3a and Additional file 1.

### Suicidal thoughts

Adolescents with overweight reported suicidal thoughts more often than their normal weight peers (original total effect OR: 1.4; 95% CI 1.2, 1.6). Adolescents who reported victimization also reported suicidal thoughts more often (*b* coefficient OR: 8.0; 95% CI 6.2, 10.3). Victimization was a significant mediator in the relationship between having overweight and suicidal thoughts (indirect effect OR: 2.1; 95% CI 1.4, 3.2). The association between overweight and suicidal thoughts, i.e. the direct effect, remained significant after the mediation effect of victimization was added to the model (OR: 1.3; 95% CI 1.1, 1.5). This is illustrated in Fig. 2b and Additional file 1.

The associations between obesity and suicidal thoughts were stronger (indirect effect OR: 4.5; 95% CI 2.3, 9.1 and direct effect OR: 1.5; 95% CI 1.1, 2.0). This is illustrated in Fig. 3b and Additional file 1.

### Discussion

Compared to their normal weight peers, adolescents with overweight or obesity reported psychosocial problems and suicidal thoughts more often. These associations were mediated by whether or not the adolescents were victimized. This indirect effect was stronger for adolescents with obesity than for those with overweight. One explanation may be that the more an individual’s body type diverges from the norm, the more they are victimized and the stronger the association with mental health problems becomes. This explanation is in line with the findings from earlier studies on this topic [36, 37].

Studies investigating the relationship between victimization and relatively uncommon physical characteristics such as having red hair, wearing glasses, disabilities and gender dysphoria demonstrated inconclusive findings [51–53]. However, our study, along with other research [27, 28, 54] shows that overweight is a physical characteristic that is related to being victimized. This may indicate that overweight is a specific trait that makes adolescents stand out negatively and thus leads to victimization. Perhaps overweight is seen as not just as a random physical trait, but as a trait with implicit social elements that may lead to stigmatization. Previous research on why overweight may lead to being bullied offered several explanations, which are all in line with the idea that overweight is perceived as a deviation from the social norm [23, 24]. As the large majority of the adolescents living in Amsterdam do not have overweight or obesity [5–7], it is plausible that normal weight is the norm among adolescents. Other studies have confirmed this by showing that overweight negatively influences social status and that pupils with overweight received fewer friendship nominations and were more disliked and more often excluded by their peers [55–57]. Such social damage can make adolescents with overweight more vulnerable to being victimized due to a lack of friends to defend them, lower self-esteem, and a lower social status among their peers [58, 59].

A recent review showed that the resulting social isolation is related to additional unhealthy behaviors: excessive food intake and decreased participation in sports and other physical activities because of increased stress and not enjoying or daring to participate in sports activities. Furthermore, overweight can lead to a low self-esteem. This can, in turn, lead to further weight gain and a vicious cycle of poor physical and social outcomes, which as a consequence could enhance the probability to become a victim [60, 61].

Besides being a victim of bullying, other potential mediators may be dissatisfaction with one’s own body [37] or reduced participation in sports and physical activity [60]. Therefore, further research using multiple mediation models is needed to better understand the relationship between overweight and negative mental health outcomes in adolescents.

Previous research demonstrated that the SDQ total difficulties score makes an excellent distinction between pupils who probably have no psychosocial problems and pupils who do, but also showed that the internal consistency of the SDQ subscales is low [39, 40]. A fruitful extension of our research would be to explore the association between overweight and different types of psychosocial problems.

### Strengths and limitations

This study is the first large-scale study examining being a victim of bullying as a potential mediator in the association between body weight and mental health problems. Our sample is representative for adolescents in Amsterdam, a multi-ethnic urban area, and likely representative for adolescents in other Dutch urban areas. Another strength is the combination of an objective report of body weight and validated self-reports of victimization and mental health problems. Being a victim of bullying and internalizing problems are less apparent to parents and teachers [62–64]. Finally, the multilevel analyses in our study allowed us to use all available information from all participants, regardless of whether a participant had information on one or two time points. A limitation is that we explored only one potential mediator in the relationship of overweight or obesity and mental health, whereas other potential mediators could also play a role. We did not distinguish between types of victimization or intensity of victimization, which may help to explain the stronger association between obesity and mental problems. Given the cross-sectional nature of our study, we were not able to infer causality. We could not infer whether overweight caused victimization and, subsequently, mental health conditions or whether adolescents with mental health conditions were more vulnerable to becoming victimized or more inclined to develop overweight. Further research is needed to examine the causal relation between overweight and mental health problems and the role that victimization and other factors may play in this relationship. Finally, we did not use a specific weight based victimization question, so it is possible that the measured victimization could be caused by other factors such as personality factors underlining victimization and mental health problems. For future research it would be of added value to use the construct of ‘weight based victimization’ as developed and studied by Puhl et al. [30, 65].

## Conclusions

Our study shows that overweight and obesity are significantly associated with mental health problems in adolescents, and that being a victim of bullying plays a role in this association. Therefore, we suggest addressing social stigma and victimization in prevention programs that promote a healthy lifestyle to improve social integration and overall quality of life [56]. For example, integrating issues relating to social stigma and victimization within programs aimed at healthy eating, physical activity and preventing overweight might result in more positive attitudes towards peers with overweight or obesity, which may lead to an improvement in their overall mental health. Without such regard for adolescents’ mental well-being, the prevention programs based narrowly on energy balance and weight control may over-emphasize the negative consequences of overweight or obesity, whereas a more positive focus on the effort and ability to control one’s own lifestyle may be more effective in promoting overall health. From a different perspective, interventions and programs aimed at preventing victimization and/or stimulating mental health should also be aware of the influence of overweight and obesity.

## Additional file

Additional file 1: Regression tables. (PDF 208 kb)

## Abbreviations

ASEBA: Achenbach System of Empirically Based Assessment
BMI: Body Mass Index
CI: Confidence Interval
DCHCR: Digital Child Health Care Registry
GGD: Amsterdam Public Health Service
IOTF: International Obesity Task Force
OR: Odds Ratio
SDQ: Strengths and Difficulties Questionnaire
YHM: Youth Health Monitor
