# Supplementary material for: Associations between overweight and mental health problems among adolescents, and the mediating role of victimization
Source: BMC Public Health. 2019 May 21;19:612. doi: 10.1186/s12889-019-6832-z (PMC6528281; doi:10.1186/s12889-019-6832-z)
Supplement: Supplementary file 1 — Regression tables. (PDF 208 kb) [file 12889_2019_6832_MOESM1_ESM.pdf]

Additional file 1 Regression tables

Addition to Figure 2A

	Regression coëfficiënt	*SE*	*P-value*	*95% CI*
**Bullying**				
**Overweight (a path)**	0.35	0.10	<.001	0.16, 0.54
Surinamese	0.48	0.13	<.001	0.22, 0.74
Turkish	0.47	0.15	.001	0.18, 0.75
Moroccan	-0.01	0.13	.926	-0.27, 0.24
Other	0.11	0.11	.307	-0.10, 0.33
Male	0.00	0.08	.977	-0.16, 0.17
**Psychosocial problems**				
**Overweight (c' path)**	0.36	0.09	<.001	0.18, 0.53
**Bullying (b path)**	2.39	0.15	<.001	2.10, 2.69
Surinamese	-0.08	0.13	.558	-0.33, 0.18
Turkish	-0.20	0.14	.154	-0.48, 0.08
Moroccan	-0.45	0.12	<.001	-0.68, -0.21
Other	-0.18	0.10	.072	-0.37, 0.02
Male	0.88	0.09	<.001	0.71, 1.05
**Indirect effect**	0.84	NA	NA	0.39, 1.32

NA=not available

Addition to Figure 2B

	Regression coëfficiënt	*SE*	*P-value*	*95% CI*
**Bullying**				
**Overweight (a path)**	0.35	0.10	<.001	0.16, 0.54
Surinamese	0.48	0.13	<.001	0.22, 0.74
Turkish	0.47	0.15	.001	0.18, 0.75
Moroccan	-0.01	0.13	.926	-0.27, 0.24
Other	0.11	0.11	.307	-0.10, 0.33
Male	0.00	0.08	.977	-0.16, 0.17
**Suicidal thoughts**				
**Overweight (c' path)**	0.27	0.08	<.001	0.12, 0.42
**Bullying (b path)**	2.08	0.13	<.001	1.82, 2.33
Surinamese	0.14	0.10	.169	-0.06, 0.35
Turkish	-0.33	0.12	.007	-0.57, -0.09
Moroccan	-0.94	0.11	<.001	-1.16, -0.72
Other	0.04	0.08	.601	-0.12, 0.20
Male	1.17	0.08	<.001	1.02, 1.32
**Indirect effect**	0.73	NA	NA	0.33, 1.15

NA=not available

Addition to Figure 3A

	Regression coëfficiënt	*SE*	*P-value*	*95% CI*
**Bullying**				
**Obesity (a path)**	0.73	0.16	<.001	0.42, 1.05
Surinamese	0.47	0.15	.001	0.18, 0.76
Turkish	0.40	0.17	.020	0.06, 0.74
Moroccan	0.12	0.14	.400	-0.16, 0.40
Other	0.20	0.12	.091	-0.03, 0.43
Male	0.01	0.09	.893	-0.17, 0.19
**Psychosocial problems**				
**Obesity (c' path)**	0.36	0.16	.029	0.04, 0.68
**Bullying (b path)**	2.49	0.17	<.001	2.16, 2.83
Surinamese	-0.08	0.15	.593	-0.36, 0.21
Turkish	-0.05	0.16	.765	-0.37, 0.27
Moroccan	-0.45	0.14	.001	-0.72, -0.18
Other	-0.15	0.11	.168	-0.36, 0.06
Male	0.80	0.09	<.001	0.62, 0.98
**Indirect effect**	1.83	NA	NA	1.03, 2.69

NA=not available

Addition to Figure 3B

	Regression coëfficiënt	*SE*	*P-value*	*95% CI*
**Bullying**				
**Obesity (a path)**	0.73	0.16	<.001	0.42, 1.05
Surinamese	0.47	0.15	.001	0.18, 0.76
Turkish	0.40	0.17	.020	0.06, 0.74
Moroccan	0.12	0.14	.400	-0.16, 0.40
Other	0.20	0.12	.091	-0.03, 0.43
Male	0.01	0.09	.894	-0.17, 0.19
**Suicidal thoughts**				
**Obesity (c' path)**	0.41	0.14	.003	0.14, 0.69
**Bullying (b path)**	2.05	0.15	<.001	1.77, 2.34
Surinamese	0.11	0.12	.359	-0.12, 0.33
Turkish	-0.17	0.14	.221	-0.44, 0.10
Moroccan	-0.86	0.13	<.001	-1.10, -0.61
Other	0.07	0.09	.462	-0.11, 0.24
Male	1.13	0.08	<.001	0.97, 1.30
**Indirect effect**	1.50	NA	NA	0.84, 2.21

NA=not available
